# Effect of a Nitrite/Nitrate-Based Accelerator on the Strength Development and Hydrate Formation in Cold-Weather Cementitious Materials

**DOI:** 10.3390/ma14041006

**Published:** 2021-02-20

**Authors:** Akira Yoneyama, Heesup Choi, Masumi Inoue, Jihoon Kim, Myungkwan Lim, Yuhji Sudoh

**Affiliations:** 1 Department of Civil and Environmental Engineering, Kitami Institute of Technology, Hokkaido 090-8507, Japan; m1952200234@std.kitami-it.ac.jp (A.Y.); m-inoue@mail.kitami-it.ac.jp (M.I.); 2 Faculty of Environmental Technology, Muroran Institute of Technology, Hokkaido 090-8585, Japan; bmjhun@mmm.muroran-it.ac.jp; 3 Department of Architectural Engineering, Songwon University, Gwangju 61756, Korea; 4 Basic Chemicals Department Chemicals Division, Nissan Chemical Corporation, Tokyo 103-6119, Japan; sudouyuuji@nissanchem.co.jp

**Keywords:** anti-freezing agent, accelerator, cold-weather concrete, calcium nitrite, calcium nitrate, strength development, pore structure, solid-state NMR, AFm phase, ettringite

## Abstract

Recently, there has been increased use of calcium-nitrite and calcium-nitrate as the main components of chloride- and alkali-free anti-freezing agents to promote concrete hydration in cold weather concreting. As the amount of nitrite/nitrate-based accelerators increases, the hydration of tricalcium aluminate (C_3_A phase) and tricalcium silicate (C_3_S phase) in cement is accelerated, thereby improving the early strength of cement and effectively preventing initial frost damage. Nitrite/nitrate-based accelerators are used in larger amounts than usual in low temperature areas below −10 °C. However, the correlation between the hydration process and strength development in concrete containing considerable nitrite/nitrate-based accelerators remains to be clearly identified. In this study, the hydrate composition (via X-ray diffraction and nuclear magnetic resonance), pore structures (via mercury intrusion porosimetry), and crystal form (via scanning electron microscopy) were determined, and investigations were performed to elucidate the effect of nitrite/nitrate-based accelerators on the initial strength development and hydrate formation of cement. Nitrite/nitrate-AFm (aluminate-ferret-monosulfate; AFm) was produced in addition to ettringite at the initial stage of hydration of cement by adding a nitrite/nitrate-based accelerator. The amount of the hydrates was attributed to an increase in the absolute amounts of NO_2_^−^ and NO_3_^−^ ions reacting with Al_2_O_3_ in the tricalcium aluminate (C_3_A phase). Further, by effectively filling the pores, it greatly contributed to the enhancement of the strength of the hardened cement product, and the degree of the contribution tended to increase with the amount of addition. On the other hand, in addition to the occurrence of cracks due to the release of a large amount of heat of hydration, the amount of expansion and contraction may increase, and it is considered necessary to adjust the amount used for each concrete work.

## 1. Introduction

When placing concrete in cold climates, it is necessary to control the temperature until the strength of the concrete at an early age reaches the required initial strength to prevent “initial frost damage” [1,2]. Currently, the most common method is to control the temperature using a heater and curing enclosure. However, this method cannot be used on steep slopes, strong winds, and narrow spaces. In these situations, it is effective to use anti-freezing agents. Generally, in concrete construction when the outside temperature is below −10 °C, the effect can be achieved by adding a large amount of anti-freezing agents [3,4,5,6,7,8].

At present, calcium nitrite (Ca(NO_2_)_2_) and calcium nitrate (Ca(NO_3_)_2_) are widely used as the main components of chloride- and alkali-free anti-freezing agents in cold-weather concreting [8,9], as well as the resistance to corrosion of reinforcing bar [10]. These accelerate the hydration reaction of the tricalcium aluminate phase (C_3_A) and tricalcium silicate phase (C_3_S) contained in cement, and contribute to the increase in hydrates such as calcium-silicate-hydrate (C–S–H; xCaO·SiO_2_·yH_2_O), ettringite (AFt; Ca_3_Al_2_[SO_4_]_3_[OH]_12_·25-27H_2_O), and monosulfate (AFm; Ca_4_Al_2_[SO_4_][OH]_12_·4-8H_2_O) at the initial stage of hydration [11,12]. Furthermore, it is known that hydrates, such as nitrite-AFm (Ca_4_Al_2_[OH]_12_[NO_2_]_2_·xH_2_O) and nitrate-AFm (Ca_4_Al_2_[OH]_12_[NO_3_]_2_·xH_2_O), are formed through reactions between the aluminate in cement and anions in the nitrite/nitrate-based accelerator [13,14,15]. These are effective for developing the initial strength of concrete and reducing the initial frost damage. However, when a large amount of nitrite/nitrate-based accelerator is used, problems are encountered, such as cracking due to an increase in expansion/contraction amount and a decrease in strength at later ages [14]. Choi et al. reported that a decrease in strength at later ages due to the consumption of a large amount of H_2_O during hydration occurs at an early age [14]. These are important problems to be solved in the use of nitrite/nitrate-based accelerators. Therefore, it is necessary to understand the correlation between hydration products and strength development.

**Figure 1:**
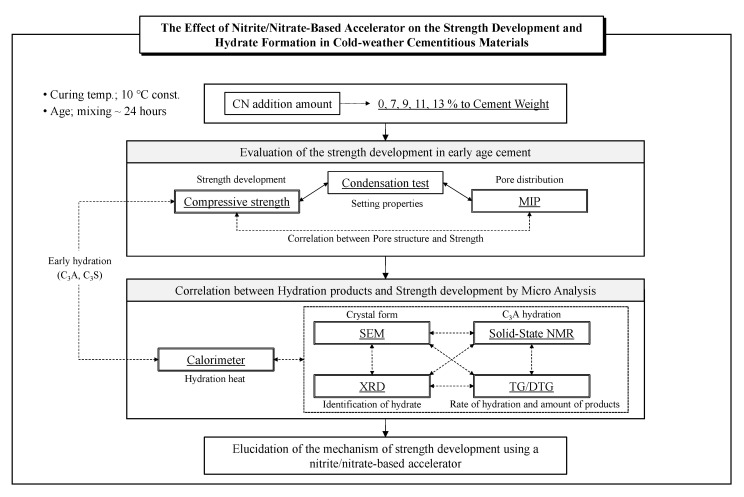
Study Flow Chart.

This study focuses on the early age (mixing ~24 h), clarifies the hydration reaction mechanism of cement when a large amount of a nitrite/nitrate-based accelerator is added, and examines the effect of NO_2_^−^ and NO_3_^−^ ions on strength development. The correlation between compressive strength properties and hydrate formation was examined by measuring the compressive strength and mercury intrusion porosimetry (MIP), and the effects of the internal structure involved in strength development were discussed. The hydrate formation process was evaluated and discussed based on the results of a calorimeter (hydration heat), thermogravimetric differential thermal analysis and differential thermogravimetric analysis (TG/DTG), X-ray diffraction (XRD), solid-state nuclear magnetic resonance (NMR), and scanning electron microscopy (SEM). Figure 1 depicts the flow chart of this study.

## 2. Experimental Overview

### 2.1. Materials and Procedures

Table 1 lists the materials used in this experiment. The cement used is a commercially available ordinary Portland cement (Taiheiyo Cement Co., Ltd., Tokyo, Japan) according to JIS R 5210 [16]. Table 2 shows the components of the anti-freezing agent (Nissan Chemical Corporation, Tokyo, Japan), and Table 3 shows the chemical composition of cement used in this experiment. An aqueous solution containing calcium nitrite (Ca(NO_2_)_2_) and calcium nitrate (Ca(NO_3_)_2_) at a concentration of 45% (hereinafter referred to as CN) was used as a component of the cold resistance accelerator. Table 4 shows the mixing ratio of the cement paste used. In this experiment, cement paste with a water-cement ratio of 0.5 was used [14,17]. In the experiment to elucidate the mechanism of strength development when a CN is added, mixing and sealed curing were performed at 10 ± 1 °C, and the curing periods were 3 h, 6 h, 12 h, and 24 h after implantation [17,18]. Normally, the amount of standard addition of existing accelerators is approximately 3~5 L per 100 kg of cement (4~7% of the cement mass) depending on the ambient temperature [17]. In this study, an addition of CN at 7% or more was defined as a “large quantity” and the experiment was carried out with CN added at five different ratios: 0%, 7%, 9%, 11%, and 13%.

According to the literature, when CN was added, Ca(NO_2_)_2_ and Ca(NO_3_)_2_ react with aluminate in cement to form a nitrite-nitrate hydrate within 24 h [14,19]. This study focuses on the early hydrate formation process from the perspective of preventing initial frost damage. Therefore, “early age” was defined as “within 24 h after mixing” [14]. The compressive strength of the cement paste was measured, and the correlation between the hydrate formation behavior and strength development was investigated. The compressive strength was measured at 24 h, and the size of the specimen was 5 cm diameter × 10 cm height. Since the pore structure greatly contributes to the strength development of the cured product, the distribution of capillary pores was confirmed by MIP. In order to understand the hydration characteristics of cement, the effect of addition of calcium nitrite on initial hydration was examined using XRD, TG/DTA, and solid-state NMR. In addition, SEM (JEOL, Tokyo, Japan) observation was performed as a method for grasping the crystal structure and pore structure of the formed hydrate, and the relationship with the strength of the cured product was considered.

### 2.2. Compressive Strength

The compressive strength was measured in accordance with JIS A 1108 [20]. The load was applied uniformly so that no impact was applied. The load speed was set to 0.6 ± 0.4 N/mm^2^ per second. Compressive strength was measured three times at each sample level, and the compressive strength results were expressed as the average of these values.

### 2.3. Mercury Intrusion Porosimetry (MIP)

The Auto-pore III 9400 Series was used to measure the MIP, and the press-fitting pressure was measured from 1.5 to 30 psia. The conditions of mercury used for measurement are as follows: Surface tension of mercury = 485 dynes/cm, contact angle = 130°, density of mercury = determined by temperature at the time of measurement. The method for preparing the sample used for the measurement is as follows: A cured sample was prepared on a dice with a side of 5 mm using a diamond cutter, hydrated with acetone, and then dried in a vacuum environment.

### 2.4. Calorimeter

The heat of hydration was measured using an isothermal calorimeter (TAM Air 8-channel; TAM Air, Tokyo, Japan), and the heat of the hydration curve was obtained. The temperature was set in a room temperature environment, and a reference (water) was created so that the heat capacity was the same as that of the measurement sample. In addition, ASTM (American Society for Testing and Materials; ASTM) 1702 was used as a reference for calculating the heat capacity. The sample used for the measurement was prepared so as to weigh 4.00 g with an electronic balance.

### 2.5. Condensation Test

The setting test was performed by the same method as that specified in JIS A 1147 [21], and the setting characteristics of the cement paste were grasped. A penetration resistance tester was used to measure the resistance when penetrating into the sample. The bleeding water generated at that time was removed. In this experiment, the time when the penetration resistance reached 3.5 N/mm^2^ was defined as the Initial setting, and the time when it reached 28 N/mm^2^ was defined as the Final setting.

### 2.6. Thermogravimetric Differential Thermal Analysis

TG/DTG was measured using TG8121 (Thermo plus EVO2 TG-DTA; Rigaku, Tokyo, Japan). The TG/DTG conditions were as follows: measuring temperature = 20~1000 °C, rising speed = 20 °C /min, atmosphere = N_2_-flow, sample weight = 15.00 mg, reference material = α-Al_2_O_3_. The method for preparing the sample used for TG / DTA measurement is as follows: Samples collected at a predetermined age are stopped hydrated with acetone, solid-liquid separated by suction filtration, and then dried in an RH11% (relative humidity; RH) environment. A sufficiently dried sample was pulverized to a particle size of 90 μm or less and used for the measurement.

### 2.7. X-ray Diffraction

XRD was performed to identify the crystal phase. Rigaku Rint2000 powder diffractometer (Tokyo, Japan) was used for the measurements. The XRD conditions were as follows: Cu-Kα radiation source = 40 kV/20 mA, scan range = 5~65 °/2θ, scan speed = 1 °/min, and step width = 0.02 °/step. The sample preparation method is as follows: A sample collected at a predetermined age was immersed in acetone to stop hydration, and suction filtration was performed for solid-liquid separation. Then, it was pulverized to a particle size of 90 μm or less and dried in an environment of 11% RH, which was used for XRD measurement.

### 2.8. Solid-State Nuclear Magnetic Resonance

^27^Al MAS NMR (Magic angle spinning) spectra were collected at 156.4 MHz on JEOL ECA-600 (magnetic field 14.1T, Tokyo, Japan) using a 3.2 mm probe. The ^27^Al NMR experiments employed a spinning speed of 16 kHz, pulse width of 1.0 µs, relaxation delay of 0.5 s, and a total of 1280 scans. Analysis of the solid-state NMR spectra was performed on a JEOL Delta NMR processing and control software (Delta 5.3.1). As the sample used for the measurement of solid-state NMR, the sample prepared in the same manner as the XRD sample was measured.

### 2.9. Scanning Electron Microscope (SEM)

In SEM, a scanning electron microscope (JSM-6510A, JEOL, Tokyo, Japan) was used to observe the microstructure on the surface of the hardened cement. The sample used for the measurement was a sample collected from a cured product, dehydrated with acetone, and dried in a vacuum environment. In addition, platinum vapor deposition was performed during the measurement.

## 3. Results and Discussion

### 3.1. Evaluation of the Strength Development in the Early Age Group

**Figure 2:**
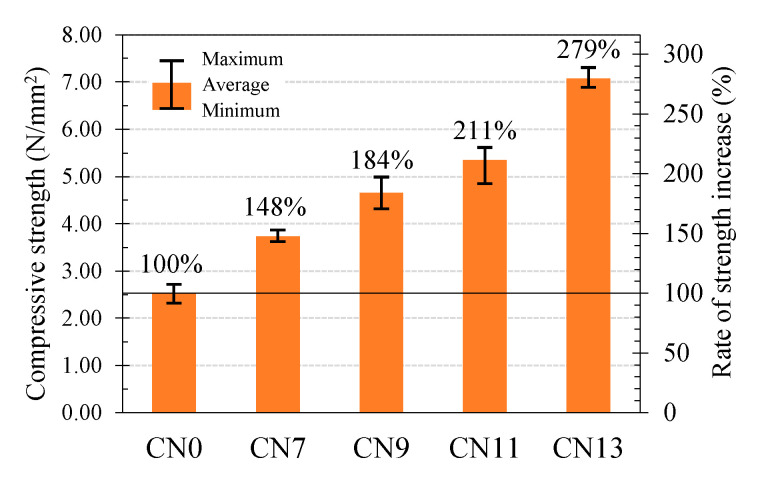
Compressive Strength.

In this study, to evaluate the effect of the early hydration progress due to the addition of CN on the strength development, the pore volume and pore diameter were measured using mercury intrusion porosimetry (MIP). Figure 2 shows the 24 h compressive strength results of the cement paste specimens cured at 10 °C for each amount of CN added. The compressive strength results in the experiment were recorded as 2.53 N/mm^2^ on CN0, 3.74 N/mm^2^ on CN7, 4.66 N/mm^2^ on CN9, 5.35 N/mm^2^ on CN11, and 7.07 N/mm^2^ on CN13, indicating a proportional correlation between the CN amount and the early compressive strength. Furthermore, the rate at which the strength increased relative to that of CN0 in 24 h was almost proportional to the amount of CN added, with 148% on CN7, 184% on CN9, 211% on CN11, and 279% on CN13.

**Figure 3:**
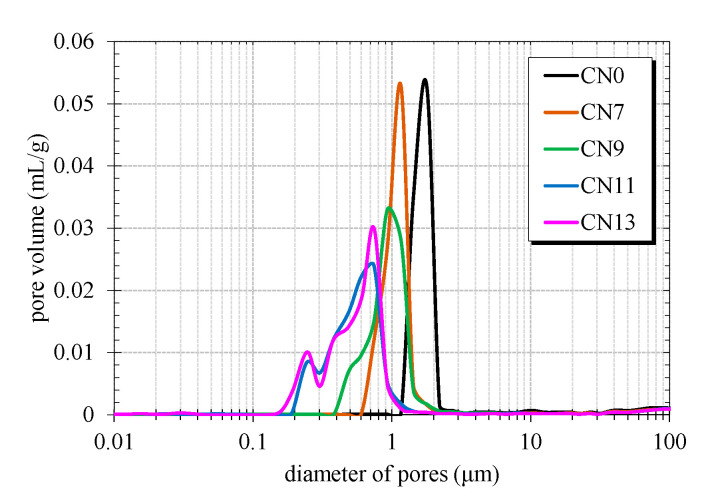
Pore Structure.

Figure 3 shows the 24 h MIP results of the cement paste specimens cured at 10 °C for each amount of CN added. From the MIP result, CN0 contains the most pores in the diameter range of 1~5 μm (coarse capillary pores). In contrast, it was confirmed that the pore size decreased as the amount of CN addition increased. In particular, CN11 and CN13 showed a distribution of pore diameters in the range of 0.1~1 µm, and pore volume tended to decrease. With regard to pore size distribution, there was no significant difference between CN11 and CN13; therefore, change of pore volume is presumed to be due to the difference in hydrate composition and rate of hydration. From the MIP results, it was confirmed that when CN was added, the pore diameter inside the hardened cement became smaller and a denser internal structure was formed.

Based on the results of these experiments, the effect of CN addition on the strength development and pore structure was discussed. In the case where CN was added, the compressive strength tended to increase in proportion to the added amount even after a curing time at 24 h. Moreover, the pore structure became denser as the hydration progressed, and the pore diameter reduced as the amount of CN added increased. The densification of the pore structure with hydration progression had a great influence on the strength development, especially at an early age, and the compressive strength of CN13 was nearly three times that of CN0. From the aforementioned results, it was found that the acceleration of hydration by the addition of CN affected the increase in cement gel and formed a fine pore structure in the cement matrix, and contributed significantly to the strength development at an early age. Additionally, the strength development might have been useful in preventing the initial frost damage.

### 3.2. Effect of Calcium Nitrite on Setting Characteristics

**Figure 4:**
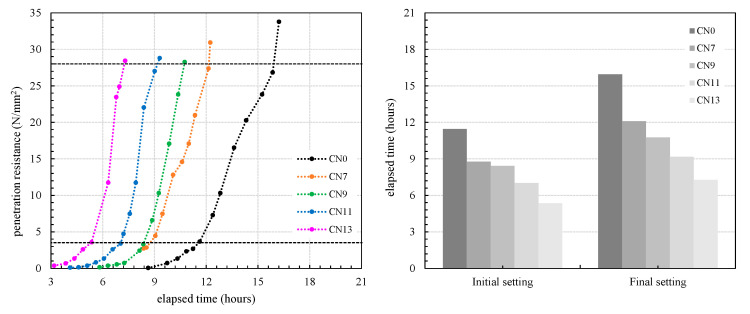
Bond strength in cement paste with different amount of CN.

In this experiment, the penetration resistance value of the cement paste prepared at a mixing temperature of 10 °C was calculated according to JIS A 1147. The time when the penetration resistance reached 3.5 N/mm^2^ was defined as the Initial setting, and the time when it reached 28 N/mm^2^ was defined as the Final setting. Figure 4 shows the relationship between the penetration resistance value and the curing time for each addition amount.

As a result of this test, the initial setting periods were 11.46 h for CN0, 8.76 h for CN7, 8.43 h for CN9, 7.01 h for CN11, and 5.35 h for CN13. Furthermore, the final setting periods were 15.95 h for CN0, 12.1 h for CN7, 10.77 h for CN9, 9.17 h for CN11, and 7.27 h for CN13, all of which were proportional to the amount added. From this tendency, it was found that the addition of CN accelerated hydration, and the effect increased in proportion to the amount of addition. In addition, it is considered that this effect increased the strength at 24 h.

### 3.3. Effect of CN Addition on the Hydration of Portland Cement

**Figure 5:**
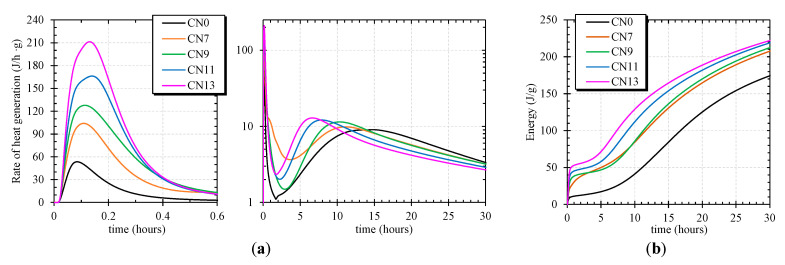
Calorimeter ((**a**): Hydration heat rate curve, (**b**): Cumulative heat of hydration).

In this experiment, the rate of heat generation was measured with a calorimeter, and the effect of CN addition on the hydration reaction was examined. Figure 5 shows the calorimeter results for each amount of CN added. First, it was confirmed that the heat of hydration rapidly increased immediately after cement made contact with water. This peak is mainly due to the heat of wetting of cement, the heat of dissolution of aluminate (Al_2_O_3_) and sulfate (SO_4_) into solution, and the heat of hydration of calcium silicate (C_3_S) (first peak) [12,22,23]. It was approximately 53 J/h·g for CN0. In contrast, it was approximately 104 J/h·g, 128 J/h·g, 166 J/h·g, and 211 J/h·g for CN7, CN9, CN11, and CN13, respectively, and the amount of heat generated tended to increase as the amount of added CN increased. After the first peak, it was confirmed that, during the dissolution of aluminate and sulfate, the hydration reaction of C_3_S was stagnant for 2~3 h, and then the second largest peak appeared (second peak) [12,22]. The second peak was attributed to the hydration of tricalcium aluminate (formation of ettringite and conversion of ettringite to the AFm phase) in cement, the heat of dissolution of the tricalcium silicate phase (C_3_S), and the heat of formation of calcium-silicate-hydrate (C–S–H), which accounts for a large proportion of the total heat of hydration [13,22]. The second peak confirmed that hydration was promoted more than in the non-added case (CN0) in all cases where CN was added. The expression time was roughly proportional to the amount of CN added, and it was 4 h for CN7, 5 h for CN9, and 7 h for CN11, and the peak for CN13 was shifted to the left side by approximately 8 h at the maximum as compared with that of CN0. The local maximum of hydration of the second peak was larger when CN was added, which was approximately 9 J/h·g for CN0 and approximately 13 J/h·g for CN13. From the calorimeter results, it was confirmed that when CN was added, the rate of hydration reaction of C_3_A and C_3_S was increased, and the amount of total heat generation was increased by the hydration of C_3_A and C_3_S at an early age [9,22,23]. The tendency of the calorific value to increase according to the amount of CN added is the same as the improvement of the compressive strength, indicating that the initial acceleration of hydration greatly contributes to the development of the initial strength.

### 3.4. Thermogravimetric Differential Thermal analysis

**Figure 6:**
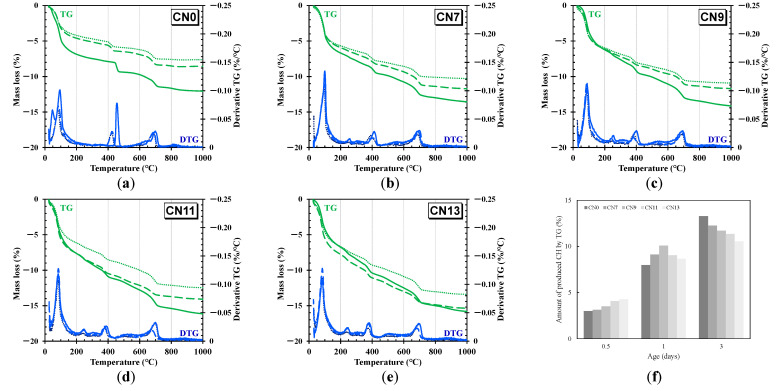
TG/DTG (TG/DTG = Green/Blue, 3 h = Dotted line, 12 h = Broken line, 24 h = Solid line). (**a**)CN0; (**b**) CN7; (**c**)CN9; (d) CN11; (**e**)CN13; (**f**) amount of CH produced by TG.

In this experiment, the thermal weight change and the derivative mass loss for each case were measured, the amount of bound water in each case was compared, and the hydrate products were identified. Figure 6 shows an example of the TG/DTG graph at curing ages of 3 h, 12 h, and 24 h and the amount of CH produced.

Balonis et al. (2011) [13] reported that when CN is added, the mass loss is in the range of 200~300 °C due to nitrite/nitrate-AFm formed by the decomposition of OH- and NO_2_-/NO_3_-groups [13]. Also in this study, in the case of the sample to which CN was added, a peak of mass reduction was observed at approximately 250~300 °C, and it was confirmed that nitrite/nitrate-AFm was produced. Moreover, the amount of mass loss at approximately 250~300 °C showed a slightly increasing trend with each curing time. In addition, the amount of mass loss in this range increases in proportion to the amount of CN added, and it is estimated that the increase in NO_2_^−^ and NO_3_^−^ ion concentration in the solution contributes to the production of nitrite/nitrate-AFm. Based on this result, it is speculated that nitrite/nitrate-AFm is generated immediately within 3 h. For other peaks, it was confirmed that the decomposition peaks increased in the range of ~100 °C in each case, and the mass loss in this range was larger in the case where CN was added. With regard to the results at ~100 °C, this decomposition peak was due to the evaporation of bound water or interphase water incorporated in AFt, AFm, and C–S–H, and it is speculated that the addition of CN accelerated the hydration of C_3_A and C_3_S at an early age [18]. The mass loss in the temperature range of 400~480 °C indicates that decomposition of CH had occurred. The amount of CH was calculated from the inflection point of the DTA curve near 400 °C. The amount of CH generated calculated from TG is as shown in Figure 6. Based on the quantitative evaluation of CH, it can be observed that the addition of CN slightly affects the formation of CH. Looking at the amount of CH produced at each age, production tended to promotion with the acceleration of hydration by the addition of CN at the age of 12 h. On the other hand, the tendency in the case of CN addition was reversed by the time the material age passed 24 h, and the tendency was inversely proportional to the addition amount at the material age of 3 days. It has been reported that CH may contribute to the formation of nitrite/nitrate-AFm when Ca(NO_2_)_2_ and Ca(NO_3_)_2_ are added [13]. Therefore, in this experiment, CH might contribute to the formation of nitrite/nitrate-AFm, which slows down the rate of CH crystal (Portlandite) precipitation, resulting in a decrease in the amount produced at an early age. In addition, the total mass loss amount increased as CN was added, indicating that the amount of bound water due to accelerated hydration of cement was increased. However, the increase in the amount of total bound water tended to increase with time in the CN0 group. In the group to which CN was added, we confirmed that the amount of bound water increased due to rapid hydration within 3 h. After that, the rate of increase in the amount of bound water between 12 h and 24 h tended to decrease compared with that of CN0. This is thought to be due to the difference in the hydration exothermic rate, and there was some correlation with the expression position of the second peak. These results indicate that, when CN is added, the hydration of cement is accelerated within 12 h and contributes greatly to the strength development in early age cement.

### 3.5. X-ray Diffraction

**Figure 7:**
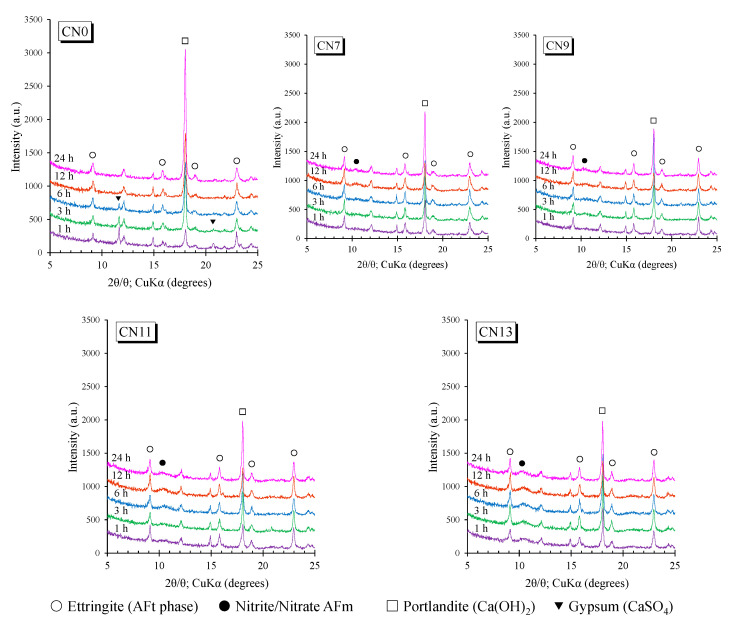
X-ray Diffraction.

In this experiment, to confirm the formation of the nitrite/nitrate-AFm reported by Balonis et al. (2011) and the effect of CN addition on the formation of other hydrates, the hydrates were identified from the XRD results. Figure 7 shows the XRD results for the diffraction angle range of 5~25° for the five cases for each curing age. Balonis et al. (2011) [13] reported that, through reaction experiments using synthetic AFm, produces nitrite (nitrate) type AFm by incorporating various anions between the layers of the AFm phase, and the ion exchange also occurs between SO_4_^2−^ and NO_2_^−^(NO_3_^−^) [13]. According to Balonis et al. (2011), nitrite-AFm and nitrate-AFm have diffraction peaks around 10~11° [13]. Based on this report, this study focused on diffraction at low angles.

At the curing age of 3 h for CN0, ettringite (2Theta/Theta= 9.1°, 15.8°, 18.9°, 22.9°) and gypsum (CaSO_4_·2H_2_O; 2Theta/Theta= 11.6°, 20.8°) peaks were visible [24,25]. In contrast, the peak of gypsum was observed in the CN0 samples but not after CN addition. From the XRD results, in the case where CN was added, it was confirmed that gypsum was rapidly consumed because of its reaction with C_3_A and ettringite. According to the contents reported by Cheung et al. (2011), it was reported that the addition of calcium nitrite markedly increased the consumption rate of gypsum and at the same time increased the production of ettringite [11]. Based on this report, in this experiment, we speculated that gypsum was rapidly consumed, and the amount of ettringite produced also increased as the amount of added CN increased. In addition, nitrite/nitrate-AFm (2Theta/Theta = 10.5°) peaks are also visible along with ettringite for all ages for the cases of CN addition. Therefore, we speculated that the addition of CN accelerated the C_3_A reaction and that the formation of AFt and nitrite/nitrate-AFm proceed simultaneously. Furthermore, the intensity of the peak for calcium hydroxide (CH; 2θ ° 18.1°) at 24 h was higher for CN0 than for the CN addition group [19,26]. It is speculated that this is because calcium hydroxide in the solution is consumed in the process of producing a large amount of ettringite and AFm by adding CN. This result is consistent with the TG/DTG results, and it is plausible that the addition of CN has an effect on the crystallinity and production of CH at an early age.

### 3.6. ^27^Al MAS NMR

**Figure 8:**
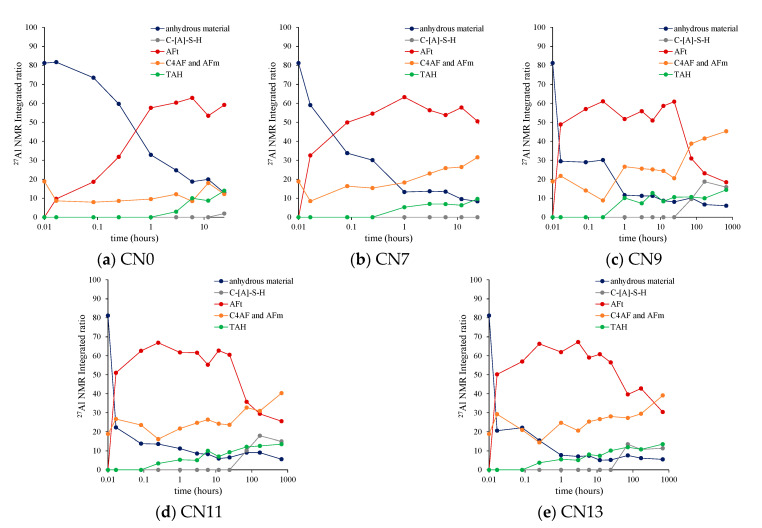
^27^Al MAS NMR integrated area ratio (mixing ~72 h).

**Figure 9:**
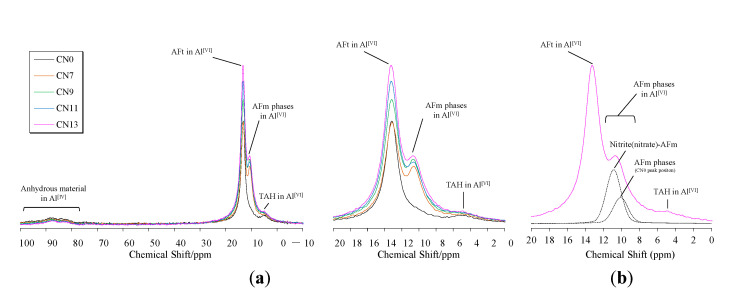
^27^Al MAS NMR spectra of each cases after 24 h. (**a**) NMR spectrum in the range of -10 to 100 ppm; (**b**) NMR spectrum derived from hydrate.

In this experiment, the progress of hydration of C_3_A and the formation of nitrite/nitrate-AFm were confirmed using ^27^Al NMR, and the NMR results were compared with the aforementioned experimental results. Figure 8 shows the solid ^27^AlNMR integrated surface integral ratio for each case from mixing ~72 h, and Figure 9 shows the results of the solid ^27^AlNMR spectrum at a material age of 24 h (−10 ppm to 100 ppm). The chemical shift region of ^27^Al NMR can be explained as follows: tetrahedral coordination (Al^[IV]^) = 50~100 ppm, pentahedral coordination (Al^[V]^) = 30~40 ppm, and octahedral coordination (Al^[VI]^) = 10~20 ppm [27,28]. Commonly, it is known that resonance in the 50~100 ppm range is due to poor crystalline structures [29,30]. The broad range of resonance from to 70~100 ppm confirmed in this study is due to the Al contained in the unhydrated cement (anhydrous material area) [19]. The NMR results reveal that the cases in which CN was added had lower peak heights and a smaller peaks area in the range of 70~100 ppm (Al^[IV]^ area) in comparison with CN0. Based on this result, the addition of CN, which accelerated the hydration of C_3_A, was confirmed, similar to the aforementioned experimental result (Section 3.1, Section 3.2, Section 3.3, Section 3.4 and Section 3.5). Furthermore, the peak in the range of 0~20 ppm (Al^[VI]^ area) is due to the resonance of Al^[VI]^ derived from the hydration product, and AFt, AFm, and third aluminate hydrate (TAH; Al(OH)_6_^3−^, O_x_Al(OH)_6−x_^(3+x)−^) peaks are mainly observed [30,31]. In addition, it has been already reported that calcium aluminoferrite (C_4_AF; Ca_2_(Al,Fe)_2_O_5_), which constitutes the clinker and overlaps with the peak of AFm, is observed at an early age [19]. In this range, ettringite (=AFt), Afm, or C_4_AF, and TAH peaks were confirmed in each case in which CN added was. Even in the case of CN0, the peaks of AFt, AFm, or C_4_AF, and TAH were confirmed as a result of the waveform separation. However, the peak position of AFm differs between CN non-added (9.6~10 ppm) and CN added (10.3~10.5 ppm), and it is considered that nitrite/nitrate-AFm was produced at an early age. In addition, the peak of the AFm phase was higher and the peak area was larger in the case where CN was added. This result confirmed that the amount of AFm produced had increased. The AFt peak tended to increase with the amount of CN added, but it was the same in all cases at the age of 24 h. It is considered that this is because almost all of the dihydrate gypsum contained in the cement was consumed. From the results of ^27^Al NMR, the addition of CN accelerates the production of AFt in addition to the production of nitrite/nitrate-AFm by the reaction of NO_2_^−^, NO_3_^−^ ions and C_3_A. Moreover, by accelerating the hydration of C_3_A, a large number of these calcium aluminate hydrates are produced, which contributes to strength development at an early age.

### 3.7. Crystal Form

**Figure 10:**
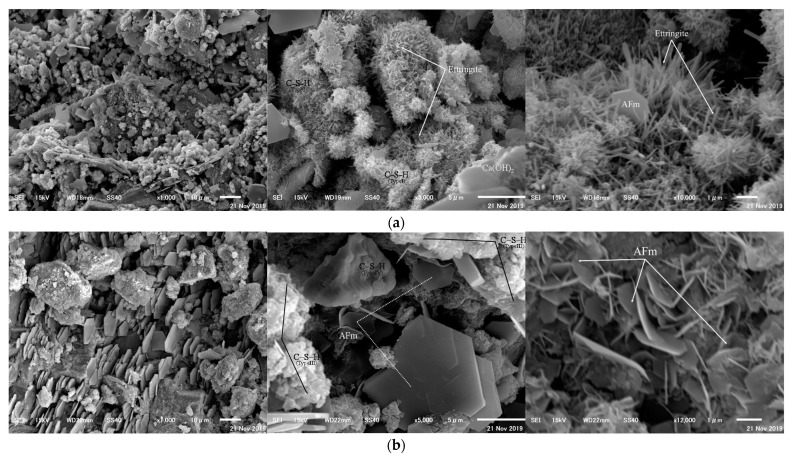
SEM images of (**a**) CN0 and (**b**) CN13.

Comparative analysis of nitrite-nitrate crystals of the hydrates produced as a result of the addition of CN was observed at 24 h for CN0 and CN13. Figure 10a,b shows the acquired SEM images. The identification of the observed crystals was determined by the crystal form, crystal size reported in previous studies, and XRD (Section 3.5) and ^27^Al NMR (Section 3.6) results in this study [13,32,33]. From the SEM images of CN0, hydration products that formed in the early stage of hydration, such as AFt, AFm(hexagonal plate-like crystals), CH, and C–S–H gel_(Type I)_, were confirmed [33]. It is known that ettringite (AFt) is formed by the reaction between C_3_A and gypsum, and is formed early in the hydration reaction in cement. Further, they greatly contribute to the strength development of cement at an early age. In the case of CN13, AFm and C–S–H gel_(Type Ⅲ, Ⅳ)_ were confirmed [33], and the crystal size was larger than that of CN0. These are usually hydrates formed after the middle stage (approximately 24 h) of the hydration reaction, and it can be seen that the addition of CN accelerated the hydration reaction in the cement [22]. These results suggest that, when CN is added, the hydration of cement in the initial stage is accelerated, and the hydrate fills the pores effectively, thereby contributing to strength development at an early age.

## 4. Conclusions

We examined the effect of internal structure on the strength development when CN was added and conducted various experimental and chemical studies to clarify the hydration mechanism at an early age. The results of the study are summarized as follows:

(1) When CN was added, the rate of ion elution from the cement clinker increased owing to the effect of NO_2_^−^ and NO_3_^−^ ions, and the rate of hydrate formation increased (especially within 12 h).
(2) The addition of CN accelerates the usual hydration reactions (C_3_A, C_3_S) that occur in the cement matrix, while additionally forming nitrite/nitrate-AFm through the reaction between C_3_A and NO_2_^−^, NO_3_^−^ ions. The hydrate generated effectively contributes to strength development by filling the pores.
(3) Nitrite/nitrate-AFm was rapidly deposited as hexagonal-plate crystals immediately after contact with water, and the production amount tended to increase as the amount of added CN increased.
(4) By adding CN, hexagonal plate-like crystals, which are presumed to be nitrite/nitrate-AFm, were confirmed in a wide range, and these are considered to contribute significantly to the strength development at an early age.
(5) Furthermore, the calorimeter, TG/DTG, and SEM results reveal that the hydration acceleration of C_3_S also contributes to the filling of pores and strength development.
(6) When nitrite/nitrate-based accelerator is added, it accelerates the hydration reaction of the initial cement and greatly contributes to the development of strength, so that problems such as delay in setting and initial frost damage in cold-weather concrete works can be improved. Furthermore, by enabling early demolding, the overall construction period can be shortened. However, there is a concern that the amount of expansion and contraction will increase due to the promotion of hydration, and there may be problems such as cracking due to heat of hydration, so it is necessary to adjust the amount used.

## Figures and Tables

**Table 1:** Properties of the materials.

Materials (Code)	Properties
Cement (C)	Ordinary Portland Cement, Density; 3.16 g/cm^3^
Anti-freezing agent (CN)	Main component; calcium nitrite, calcium nitrate (45% water solution), Density; 1.43 g/cm^3^

**Table 2:** Properties of the anti-freezing agent (nitrite-nitrate based accelerator; CN).

Component	Component Ratio	Density of Aquarius Solution (g/cm^3^)	pH Aquarius Solution
Ca(NO_2_)_2_	23.02%	1.43	9.3
Ca(NO_3_)_2_	22.81%		

**Table 3:** Chemical composition of cement.

	Chemical Composition (%)								
Ordinary Portland Cement	SiO_2_	Al_2_O_3_	Fe_2_O_3_	CaO	MgO	SO_3_	CaSO_4_	Ig.loss	Alkali Content
	21.4	5.5	2.8	64.3	2.1	1.9	-	0.56	0.25

**Table 4:** Properties of the cement-paste mix.

Type	W/C (%)	Unit Content (kg/m^3^)		Anti-Freezing Agent (C × %)
		W	C	CN
CN0	50	612	1225	0
CN7				7
CN9				9
CN11				11
CN13				13

Note: W/C; water-cement ratio, (C × %).

## Data Availability

Data available on request due to restrictions eg privacy or ethical.
